# Characterization and validation of long noncoding RNAs as new candidates in prostate cancer

**DOI:** 10.1186/s12935-020-01615-y

**Published:** 2020-11-01

**Authors:** Shengyang Ge, Yuanyuan Mi, Xiaojun Zhao, Qingfeng Hu, Yijun Guo, Fan Zhong, Yang Zhang, Guowei Xia, Chuanyu Sun

**Affiliations:** 1 Department of Urology, Huashan Hospital, Fudan University, 12 Central Urumqi Rd, Shanghai, 200040 P. R. China; 2 Department of Urology, Affiliated Hospital of Jiangnan University, Hefeng Rd, Wuxi, 214000 PR China; 3 Department of Clinical Immunology, Shanghai Center for Clinical Laboratory, 528 Hongshan Rd, Shanghai, 200126 P. R. China; 4 Department of Urology, Jing’an District Central Hospital, Fudan University, 259 Xikang Rd, Shanghai, 200040 P. R. China; 5 Department of Systems Biology for Medicine, and Institutes of Biomedical Sciences, Shanghai Medical College, Fudan University, 130 Dongan Rd, Shanghai, P. R. China

**Keywords:** Prostate cancer, lncRNA, Microarray

## Abstract

**Background:**

Long noncoding RNAs (lncRNAs) have been proved to be an important regulator in gene expression. In almost all kinds of cancers, lncRNAs participated in the process of pathogenesis, invasion, and metastasis. Meanwhile, compared with the large amounts of patients, there is rare knowledge about the role of lncRNAs in prostate cancer (PCa).

**Material/Method:**

In this study, lncRNA expression profiles of prostate cancer were detected by Agilent microarray chip, 5 pairs of case and control specimens were involved in. Differentially expressed lncRNAs were screened out by volcano plot for constructing lncRNA-miRNA-mRNA central network. Then, the top ten up-regulated and down-regulated lncRNAs were validated by qRT-PCR in another 5 tumor specimens and 7 para-cancerous/benign contrasts. Furthermore, we searched for the survival curve of the top 10 upregulated and downregulated lncRNAs.

**Results:**

A total of 817 differentially expressed lncRNAs were filtered out by the criteria of fold change (FC) and t-test p < 0.05. Among them, 422 were upregulated, whereas 395 were downregulated in PCa tissues. Gene ontology and KEGG pathway analyses showed that many lncRNAs were implicated in carcinogenesis. lnc-MYL2-4:1 (FC = 0.00141, *p* = 0.01909) and NR_125857 (FC = 59.27658,* p* = 0.00128) had the highest magnitude of change. The subsequent qPCR confirmed the expression of NR_125857 was in accordance with the clinical samples. High expression of PCA3, PCAT14 and AP001610.9 led to high hazard ratio while low expression of RP11-279F6.2 led to high hazard ratio.

**Conclusions:**

Our study detected a relatively novel complicated map of lncRNAs in PCa, which may have the potential to investigate for diagnosis, treatment and follow-up in PCa. Our study revealed the expression of NR_125857 in human PCa tissues was most up-regulated. Further studies are needed to investigate to figure out the mechanisms in PCa.

## Introduction

Prostate cancer (PCa) is one of the most common malignancy in males, like in the United State, it causes estimated 191,930 cases and 33,330 deaths in 2020 [1]. PCa ranks the fifth leading cause of cancer death worldwide [2]. With the development of economy and society, China is experiencing a transition stage from a developing country into a developed country, named as “westernized lifestyle-related cancer”, so we can easily find that there is an ascendant tendency in the incidence rate of PCa [3]. Many conventional high-risk factors have been concerned with the period of tumorigenesis, invasion and metastasis of PCa, including genetic, environmental and life-style factors [4, 5].

PCa is normally hormone-dependent at diagnosis indicating androgen receptor (AR) signaling is a distinctive feature in this disease [6]. The AR is a ligand-activated transcription factor typically responsive to the androgen testosterone and dihydrotestosterone [7]. Androgen deprivation therapy (ADT), urological surgery or chemical castration, is a standard treatment used in recurrent PCa [8]. Even though most of patients with PCa are treated successfully, a significant proportion of patients would develop castration-resistant prostate cancer (CRPC), many of which further block the androgen axis [9]. Recent studies revealed that the frequency of AR-null CRPC is increasing, because of the application of more effective AR antagonists such as enzalutamide and abiraterone [10]. Since the effort of urologists for the patient in the end stage of this disease is limited, it is imperative for the scientists to progress effective biomarkers for very early detection and active target for clinical treatment.

Up to now, protein PSA (Prostate Specific Antigen) is the only biomarker used in clinical practice. Folk with high risks in PCa were screened out by measuring expression of PSA in the blood. The US Preventive Services Task Force (USPSTF) doubted the reliability and application of PSA by retrospective study [11]. For men aged from 55 to 69 years, the decision to undergo periodic PSA-based screening for PCa should be well assessed based on their particlular clinical characteristics like family history [12]. Therefore, it gives a few patients a limited potential profit of prognosis through screening PSA [13]. Many men will suffer potential harms of screening, including false-positive results that entail extra testing and even, invasive prostate biopsy, to separate them from the real patients; overdiagnosis and overtreatment; and it may arouse a lot of treatment complications, such as incontinence and erectile dysfunction, which wastes unnecessary time, influences the normal life and lowers the quality of life [14]. Clinicians should not screen men who do not express a preference for screening (C recommendation) [15–17]. The USPSTF recommends against PSA-based screening for PCa in men 70 years and older (D recommendation) [11, 18, 19]. As there is progressively amounts of argument and distrust about the specificity and sensitivity of PSA, it is essential for us to advance more dependable biomarkers for early screening of PCa.

Non-coding RNAs (ncRNAs) are a class of RNA molecules that lack protein-coding potential. Accumulating genomic and transcriptomic sequencing results have revealed that only small proportion of the human genome is transcribed into protein-coding mRNAs, whereas the majority of the genome is transcribed into ncRNAs [20, 21]. Amongst the classes of ncRNAs, long noncoding RNAs (lncRNAs) are a class of transcripts longer than 200 nucleotides with limited protein coding potential [22]. Unlike proteins, ncRNAs function cannot currently be inferred from sequence or structure, with the diversity of long ncRNAs described to date precluding simple generalizations [23]. LncRNAs regulate local protein-coding gene expression at the level of chromatin remodeling, transcriptional control and post-transcriptional processing, which suggests that RNA has continued to evolve and expand alongside proteins and DNA and indicate they have multiple functions in a wide range of biological processes, such as proliferation, apoptosis, or cell migration [24, 25]. Various of transcriptomics studies showed that some kind of lncRNA dysregulated in different cancers, including neuroblastoma, pancreatic ductal adenocarcinoma, lung cancer and other cancers through corresponding miRNAs [26–29]. Moreover, this abnormal phenomenon are also detected in circulating blood and/or urine [30–32]. LncRNA is a novel class of potential biomarkers and therapeutic targets for the treatment of cancer [33].

Nevertheless, the function of most lncRNAs is still unknown. A growing amount of evidence has showed that lncRNAs play a vital role in the progression of PCa [34]. Especially, the expression levels and potential roles of lncRNAs in PCa are needed to further investigated [35]. Herein, we combined our analysis of RNA-seq datasets, from 5 patient samples, including PCa and adjacent benign prostate tissue with the other investigation to exploit and corroborate differentially expressed lncRNA connected with PCa. After we detected the dysregulated lncRNAs from transcriptome profiles, we validated these lncRNAs from RNA-seq with qRT-PCR using another 5 tumor specimens and 7 para-cancerous/benign contrasts from prostate biopsy.

## Material and method

### Tissue samples

A group of 5 pairs of PCa and matched non-tumor normal tissues were collected from Huashan Hospital, Fudan University. To deep confirm, another cohort of prostate tissues were obtained from prostate needle biopsies in Huashan Hospital, Fudan University. Our study was permitted by the ethics committee of Huashan Hospital, Fudan University (ethics approval no. 2011-009) and written informed consent was obtained from all patients. All tissue was histologically identified by pathological section. If diagnosed as prostate adenocarcinoma, the Gleason score, PSA value, TNM stage and recurrence were according to the NCCN guideline [36]. Otherwise, the tissues were recognized as normal contrast. A subset of patients had matched PCa tissues and normal tissues available for qPCR. The initial screening step (Table 1) was conducted with microarray chip assay. Another cohort screening information which was considered as the validation of the expanded clinical samples (Table 2) was listed with the qPCR.

**Table 1:** The main clinical information of patients with PCA included in our study

NO	Gender	Age (Years)	Histological type	Initial total PSA	Gleason score	TNM stage
12	Man	64	Adenocarcinoma	9.39	3 + 4	T2cN0M0
34	Man	50	Adenocarcinoma	15.84	4 + 3	T3bN0M0
56	Man	62	Adenocarcinoma	14	4 + 3	T3bN0M1
78	Man	54	Adenocarcinoma	9.13	4 + 3	T2cN0M0
910	Man	62	Adenocarcinoma	54.66	5 + 4	T3bN1M1

**Table 2:** The clinical characteristics of extended samples by prostate biopsy for qPCR included in our study

NO	Gender	Age (years)	Histological type	Initial total PSA	Gleason score	Proportion of Cancer tissue
001	Man	62	Adenocarcinoma	10.44	3 + 4	80%
002	Man	51	Adenocarcinoma	8.08	4 + 5	70%
003	Man	53	Adenocarcinoma	9.11	4 + 4	30%
004	Man	54	Adenocarcinoma	8.48	3 + 3	5%
005	Man	55	Adenocarcinoma	11.84	4 + 3	60%
006	Man	63	Normal tissue	10.92	/	/
007	Man	78	Normal tissue	9.46	/	/
008	Man	50	Normal tissue	9.56	/	/
009	Man	63	Normal tissue	12.71	/	/
010	Man	72	Normal tissue	10.82	/	/
011	Man	77	Normal tissue	8.33	/	/
012	Man	58	Normal tissue	9.21	/	/

### RNA extraction and purification

Total RNA of tissue specimen was extracted and purified using mirVana™ miRNA Isolation Kit (Cat#AM1561, Ambion, Austin, TX, US) following the manufacturer’s instructions and checked for a RIN number to inspect RNA integration by an Agilent Bioanalyzer 2100 (Agilent technologies, Santa Clara, CA, US).

### RNA labeling

rRNA was amplified and labeled by Low Input Quick Amp WT Labeling Kit (Cat.# 5190–2943, Agilent technologies, Santa Clara, CA, US), following the manufacturer’s instructions. Labeled cRNA were purified by RNeasy mini kit (Cat.# 74,106, QIAGEN, GmBH, Germany).

### Array hybridization

Each slide was hybridized with 1.65 μg Cy3-labeled cRNA using Gene Expression Hybridization Kit (Cat.# 5188-5242, Agilent technologies, Santa Clara, CA, US) in Hybridization Oven (Cat.# G2545A, Agilent technologies, Santa Clara, CA, US), according to the manufacturer’s instructions. After 17 h hybridization, slides were washed in staining dishes (Cat. # 121, Thermo Shandon, Waltham, MA, US) with Gene Expression Wash Buffer Kit (Cat.# 5188-5327, Agilent technologies, Santa Clara, CA, US), followed the manufacturer’s instructions. Differentially expressed lncRNAs were analyzed with independent samples t-test. LncRNAs with ≥ 2.0 fold-changes (FC) and *p* < 0.05 were selected as lncRNAs with significant differential expression.

### Data acquisition

Slides were scanned by Agilent Microarray Scanner (Cat#G2565CA, Agilent technologies, Santa Clara, CA, US) with default settings, Dye channel: Green, Scan resolution = 3 μm, PMT 100%, 20bit. Data were extracted with Feature Extraction software 10.7 (Agilent technologies, Santa Clara, CA, US). Raw data were normalized by Quantile algorithm, Limma packages in R.

### Bioinformatics analysis

LncRNA targets identified with profiling data were subjected to Gene Ontology (GO) and Kyoto Encyclopedia of Genes and Genomes (KEGG) pathway analyses based on their correlated mRNAs using GO (https://www.geneongoloty.org/) and KOBAS software (KEGG Orthology-Based Annotation System, https://www.kegg.jp/), [37, 38]. The differentially expressed lncRNAs-targeted miRNAs were sought and predicted by miRanda software (https://miranda.org.uk/) coupled with statistical analysis. The lncRNAs expression profile microarray chip assay, besides data and bioinformatics analysis were carried out by Shanghai Biotechnology Corporation (Shanghai, China).

### qPCR analysis

Total RNA from another normal tissues (9 samples) and PCa tissues (7 samples) sustained by pathology after perineal prostate biopsy guided by ultrasound was prepared by Trizol Isolation Reagent (Invitrogen). Dimethylcarbinol, ethanol and trichloromethane were of analytic grade. DNase I, SYBR Green Realtime PCR Master Mix Plus and the ReverTra Ace qPCR RT Kit are from Toyobo Co. Japan. 2 μg RNA was extracted from each sample was used for cDNA preparation. The reverse transcription kit steps are strictly followed to transcribe to cDNA. cDNA was used as template, and hGAPDH as internal parameter. The primer concentration was set as 0.4 μmol/L. Three parallel samples were set for each sample, tested as 15 μl system used for amplification. For qPCR solution, THUNDERBIRD SYBR qPCR Mix (Toyobo, Osaka, Japan) was utilized. qPCR was performed on the LightCycler 96 (Roche, Indianapolis, IN, USA) following the instruction. The reaction conditions of qPCR were: pre-denaturation at 95 °C for 3 min, denaturation at 95 °C for 15 s, annealing at 60 °C for 15 s, extension at 72 °C for 20 s and totally 40 cycles. Assay numbers got involved in the top 10 up-regulated and down-regulated expression of lncRNAs and GAPDH, respectively. The sequences of primer are listed in the Table 3. Differentiated gene expression was calculated by the comparative Ct method.

**Table 3:** The list of the primer sequence for the top 10 up-regulated and down-regulated lncRNAs

The accession of lncRNA	The primer sequence
hGAPDH-Q-F	TCAAGGCTGAGAACGGGAAG
hGAPDH-Q-R	TCGCCCCACTTGATTTTGGA
NR_125857-F	CCCATCCTCATTTGGTGCTG
NR_125857-R	CAACAGACAACACGAGGCAG
NR_015342-F	GAAGCACCTCGCATTTGTGG
NR_015342-R	TTTCTCAAACCGCCTGATGC
NR_109832-F	TCCGTCTCCTGCATGTCCTTGG
NR_109832-R	ACCTTCACCCTCCAGCCACAG
ENST00000412654-F	AGCATGGTCCCCAATGTAGC
ENST00000412654-R	CCACCCATGAGGCGTAATCA
lnc-AC110080.1–5:1-F	CCATCTCCTGCAAGTCTAGTCA
lnc-AC110080.1–5:1-R	TGCCACTGAACCTATCTGGC
ENST00000415820-F	GAGGGTAGATGGAGCATCGC
ENST00000415820-R	TTCCAGTTCTTTGCTCCGCA
ENST00000558010-F	TGCCCGTAATCCCTTTGTCC
ENST00000558010-R	ATTTGGTGCCGTGTGCTAGA
ENST00000365110-F	TTGCACGTTGTTGGAGCTTG
ENST00000365110-R	AATTTGCCCCTCACGTAGCA
NONHSAT072254-F	CAGGGCCAGTCAGAGTCTTTC
NONHSAT072254-R	CCACTGAACCTGTCTGGGATG
NONHSAT072236-F	CAGGGCCAGTCAGAGTCTTTC
NONHSAT072236-R	CCACTGAACCTGTCTGGGATG
ENST00000424251-F	TGGCATGAGCAAACTTGGGC
ENST00000424251-R	CAGTGCCAATAACGGCCACA
lnc-TACC2-3:1-F	ACGCCTGCATCTTCACAAAG
lnc-TACC2-3:1-R	TCAAAGCTACACAATGCGGG
NR_125859-F	TTTGCCCATAAGTCTCCCTGG
NR_125859-R	TTCCAAGCCAGCGTTTTCAC
NONHSAT136589-F	TGCTGGCTGCTCTGAACTAAA
NONHSAT136589-R	TCCAGCTTTTAGGCACACACA
lnc-CHST2-2:3-F	TGCAGACAAGTGTGTATGAGT
lnc-CHST2-2:3-R	CTGTCTGCTAACAAAGGGTTCA
lnc-PDCD11-5:1-F	AATCCCATCAGGCGTAGGG
lnc-PDCD11-5:1-R	GCAGAAATCACACCCAGGTTC
lnc-PTEN-11:1-F	TGCCAGTCTCTAGGTCCCTG
lnc-PTEN-11:1-R	AGACGCCAGGCTCCCAA
lnc-MID1-4:1-F	CAGAGCAAGGCACCCACTAA
lnc-MID1-4:1-R	CCCACGACTGCTCCAAAGTA
lnc-C19orf73-1:1-F	ACTGCGACACAGCGGTA
lnc-C19orf73-1:1-R	GGAGCACGTTTATTCAGAGAAAT
lnc-MYL2-4:1-F	TATTGTTCCTGGGCTGCAGA
lnc-MYL2-4:1- R	GGAGAACACGTTGGAGTTGG

^*^-F presented the forward primer while -R presented the reverse primer

### The co-expression network of lncRNA-miRNA-mRNA

Spearman correlation was calculated between the abundance of each lncRNA against each miRNA with the criteria of relative expression levels. Once the predicted pairs of lncRNA-miRNA relation were determined, and further filtered by comparison with the theoretical databases. The theoretical databases included ENCORI, lncBase, miRcode for lncRNA-miRNA relations and miRcode, ENCORI, TarBase, miRTarBase, miRDB, miRanda, miRecords for miRNA-mRNA relations. For Agilent chip GPL22120, adopt multiple IDs from different sources, should be correspond to one unique ID, and we map the all IDs in GPL22120 to RNA Central (https://www.rnacentral.org/, v14) [39]. All the lncRNA ID listed in figure were started by “URS” which was the acronym of Unique RNA Sequence and combined with 10 numbers and/or English letters.

### Survival Curve Analysis

We used the GEPIA (Gene Expression Profiling Interactive Analysis, https://gepia.cancer-pku.cn/) as tool to search for the survival curve of the top 10 upregulated and downregulated lncRNAs original gene [40].

### Statistical analysis

All data are shown as mean ± standard deviation (SD). Statistical significance was determined using Student's t-test by SPSS 13.0 and Graphpad Prism 5. *p* < 0.05 was considered statistically significant.

## Result

### LncRNAs expression profiles in PCa

**Fig. 1:**
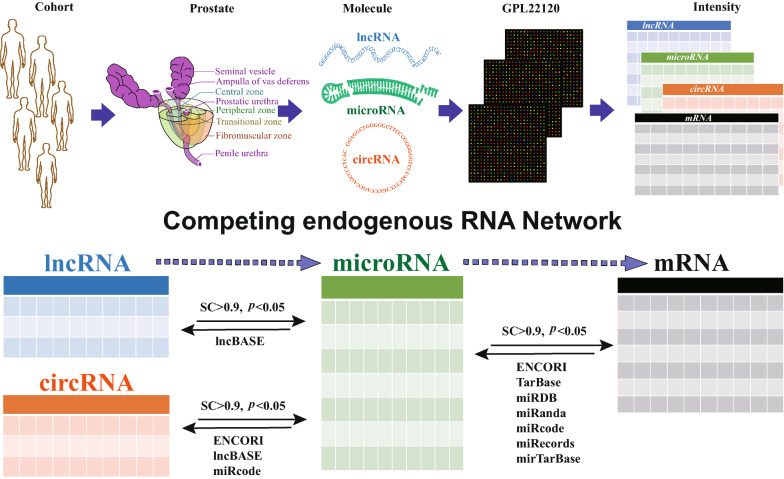
The diagram of data processing. In this study, lncRNA expression profiles were screened in PCa, by using five pairs of clinical specimens in PCa and matched non-PCa tissues with lncRNA chip GPL22120. The abundance of each lncRNA against each miRNA was calculated using Spearman correlation, and then filtered by comparison with the theoretical databases. The theoretical databases included ENCORI, lncBase, miRcode for the relations of lncRNA-miRNA and miRcode, ENCORI, TarBase, miRTarBase, miRDB, miRanda, miRecords for the relations of miRNA-mRNA

**Fig. 2:**
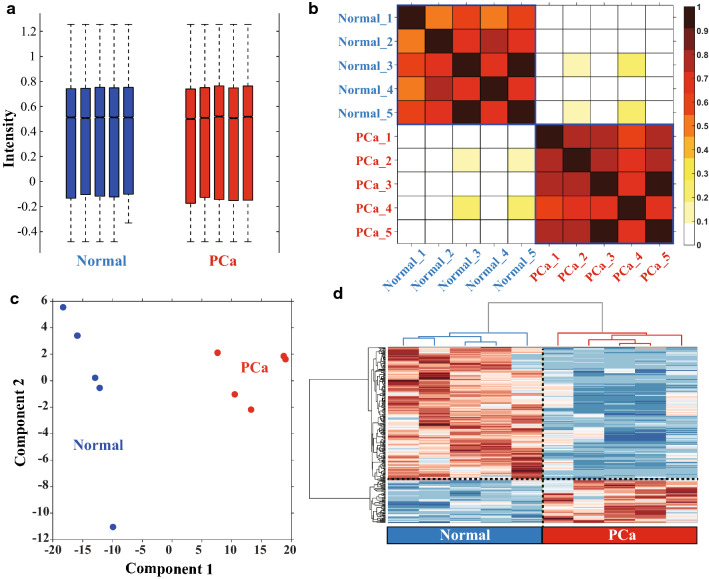
Global view of all lncRNAs expression in PCa tissues compared to paired non-PCa tissues. **a** Boxplot. The medians between samples were roughly flat, and the ranges of expressions were similar. **b** Sample correlation matrix. The correlation coefficient within the groups was significantly higher than that between the groups, which indicated the larger differences between PCa tissues and paired non-PCa tissues. **c** Principal component analysis. The difference between normal and tumor was large in the first principal component, but slightly in the second principal component, which showed there was a big difference between the samples. **d** Hierarchy Clustering Analysis. Repeated samples are clustered together, indicating the repeatability of samples and the differences between samples. The black dotted line divides lncRNAs into two categories: above the line, it presented the part of high expression in non-PCa tissues and low expression in PCa, and below the line, it presented the part of high expression in PCa and low expression in non-PCa tissues. Overall, through a variety of global analysis, we concluded that our tissue samples used in our study presented good reproducibility and the large differences between groups.

**Fig. 3:**
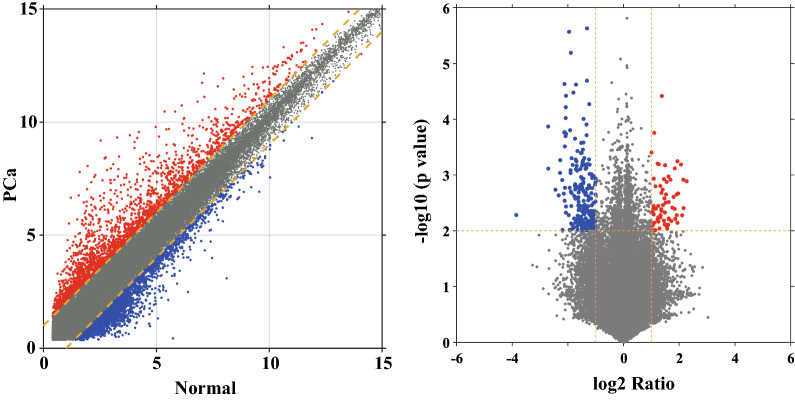
Selection of differentially expressed lncRNAs. The scatter plots and volcano plots exhibited the differentially expressed lncRNAs in PCa tissues compared to paired non-PCa tissues. The left figure presented the scatter plots while the right figure presented the volcano plots

The microarray screening identified 68,424 lncRNAs in PCa, non-PCa or both tissues. As illustrated in Fig. 1, totally, 817 lncRNAs were differentially expressed between PCa tumor and paracancerous tissues ($\mathrm{FC}\ge 2.0\;and\;p<0.05$): among which 422 were upregulated, and the remaining 395 were downregulated in PCa tissues. The magnitude of FC was the highest for NR_125857 for upregulated lncRNAs ($FC=59.27658, p=0.00128$) while it was the lowest for lnc-MYL2-4:1 in downregulated lncRNAs ($\mathrm{FC}=0.00141, p =0.01909$). Hierarchical clustering (Fig. 2D), volcano plot (Fig. 3), and scatter plots (Fig. 3) shown that the different expression profiles of lncRNAs between PCa and non-PCa tissues were diverse. The top each twenty up- and down-regulated lncRNAs were listed in Table 4.

**Table 4:** Top each ten up- and down-regulated lncRNAs and corresponding gene information of lncRNAs

Accession	*P*-values	Fold change	Regulation	Chromosome		Strand	Gene symbol	Associated gene description
NR_125857	0.0012796	59.276583	up		chr6	+	EVADR	−
NR_015342	0.003856	28.092424	up		chr9	+	PCA3	prune homolog 2 (Drosophila)
NR_109832	0.0087905	21.726513	up		chr22	+	PCAT14	−
ENST00000412654	0.0026089	19.731117	up		chr9	+	PCA3	prune homolog 2 (Drosophila)
lnc-AC110080.1–5:1	0.0214716	17.480829	up		chr2	−	–−	−
ENST00000415820	0.0175281	16.656541	up		chr21	−	AP001610.9	−
ENST00000558010	0.0150573	15.600412	up		chr15	−	RP11-279F6.2	−
ENST00000365110	0.0289324	13.988218	up		chr11	+	SNORA62	−
NONHSAT072254	0.0075766	12.035524	up		chr2	−	–−	−
NONHSAT072236	0.0072588	11.671845	up		chr2	−	–−	−
ENST00000623273	0.0117887	0.2264097	down		chr5	−	CTB-174D11.3	slit homolog 3 (Drosophila)
ENST00000424251	0.0143991	0.2204195	down		chrX	+	RP1-146A15.1	interleukin 1 receptor accessory protein-like 1
lnc-TACC2-3:1	0.0146426	0.2146128	down		chr10	+	–−	transforming, acidic coiled-coil containing protein 2
NR_125859	0.0322068	0.2054874	down		chr6	+	LOC101928540	filamin A interacting protein 1
NONHSAT136589	0.0022274	0.1790319	down		chrX	−	–−	dystrophin
lnc-CHST2-2:3	0.023774	0.1771025	down		chr3	+	–−	−
lnc-PDCD11-5:1	0.000183	0.1635704	down		chr10	+	–−	neuralized E3 ubiquitin protein ligase 1
lnc-PTEN-11:1	0.0032078	0.1472261	down		chr10	+	–−	-
lnc-MID1-4:1	0.00866	0.1373993	down		chrX	−	–−	Rho GTPase activating protein 6
lnc-C19orf73-1:1	0.034911	0.030913	down		chr19	−	–−	histidine rich calcium binding protein
lnc-MYL2-4:1	0.0190929	0.0014053	down		chr12	−	–−	myosin, light chain 2, regulatory, cardiac, slow

### Bioinformatics analysis of differential expressed lncRNAs

**Fig. 4:**
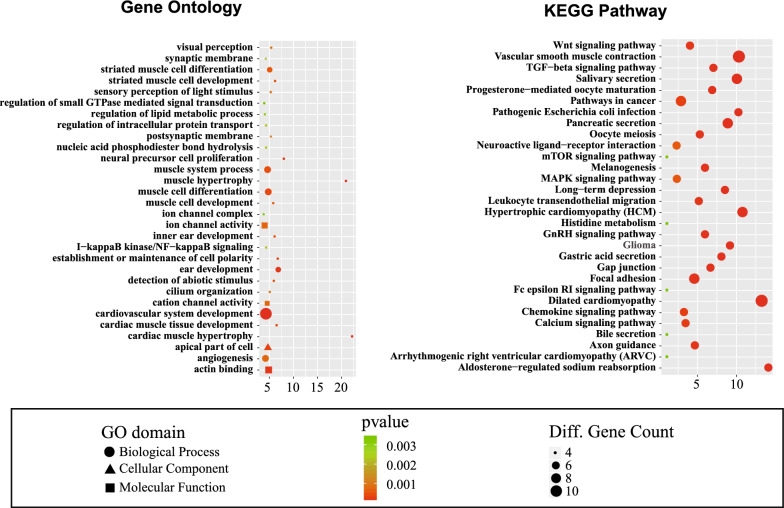
Results of Gene Ontology and KEGG pathway analysis. **a** Top 30 classes of GO enrichment terms. **b** Top 30 classes of KEGG pathway enrichment terms

Top each twenty up- and down-regulated lncRNAs and corresponding gene information of lncRNAs were shown in Table 4. Moreover, each Top 30 enrichments about GO and KEGG analyses suggested that these differentially expression lncRNAs were relevant to several vital physiological processes, such as cardiac muscle hypertrophy, muscle hypertrophy, neural precursor cell proliferation, establishment or maintenance of cell polarity, cardiac muscle tissue development, striated muscle cell development, muscle cell development, actin binding, and postsynaptic membrane. Intriguingly, most of them are associated with the muscle tissue development, including cardiac muscle and striated muscle, which may hint the reorganization of the excellular matrix on behalf of the smooth muscle surrounding the PCa. Moreover, the upregulation of the neuron formation shows the nerve paracrine factor involving in the tumorigenesis. Except for the famous pathways, such as TGF-$\upbeta$, Wnt, MARK and mTOR that have been proven to be closely correlated to proliferation, invasion and metastasis in PCa, astonishingly, the pathway of aldosterone-regulated sodium reabsorption, dilated cardiomyopathy, hypertrophic cardiomyopathy, pathogenic Escherichia coli infection and vascular smooth muscle contraction also implies the revegetation of smooth muscle may interfere with the microenvironment of PCa. Additionally, the pathogenic Escherichia coli infection may link to the common urinary disease, prostatitis, which also causes the tissue recovery (Fig. 4).

### Validation of qPCR

**Fig. 5:**
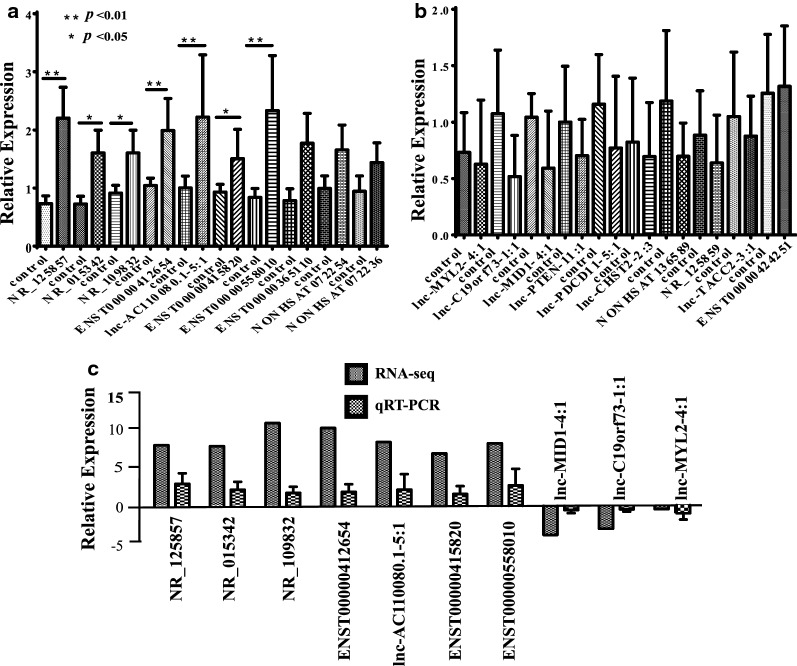
The outcomes of subsequent verification tests by qPCR. **a** The relative expression of top ten upregulated lncRNAs in qPCR. There were significant statistic differences in NR_125857, NR_015342, NR_109832, ENST00000412654, lnc-AC110080.1–5:1, ENST00000415820, ENST00000558010 (*p* < 0.05). **b** The relative expression of top ten downregulated lncRNAs in qPCR. There was no significant statistic difference between them (*p* > 0.05), which may be due to the small sample size. **c** Confirmation of the expression patterns of lncRNAs by comparing the results of qRT-PCR and original array outcomes of RNA-seq. Top 7 up-regulated lncRNAs and top 3 down-regulated lncRNAs

The outcome of qPCR showed significant statistic differences in NR_125857, NR_015342, NR_109832, ENST00000412654, lnc-AC110080.1–5:1, ENST00000415820, ENST00000558010 ($p\hspace{0.17em}<\hspace{0.17em}0.05$) in PCa tissues (n = 5) compared to normal prostate tissues (n = 7) while there was no statistic difference of all the top ten downregulated lncRNA expression (Fig. 5a, b). These results indicated that the expression of NR_125857, NR_015342, NR_109832, ENST00000412654, lnc-AC110080.1–5:1, ENST00000415820, ENST00000558010 were up-regulated in PCa and it revealed whether the downregulation of ENST00000424251, lnc-TACC2-3:1, NR_125859, NONHSAT136589, lnc-CHST2-2:3,lnc-PDCD11-5:1,lnc-PTEN-11:1, lnc-MID1-4:1, lnc-C19orf73-1:1, lnc-MYL2-4:1 was still doubted. Compared with the original outcomes of RNA-seq array, the relative expression of qPCR in the additional samples showed consistency in figure (Fig. 5C).

### Survival analysis of differential expressed lncRNAs

**Fig. 6:**
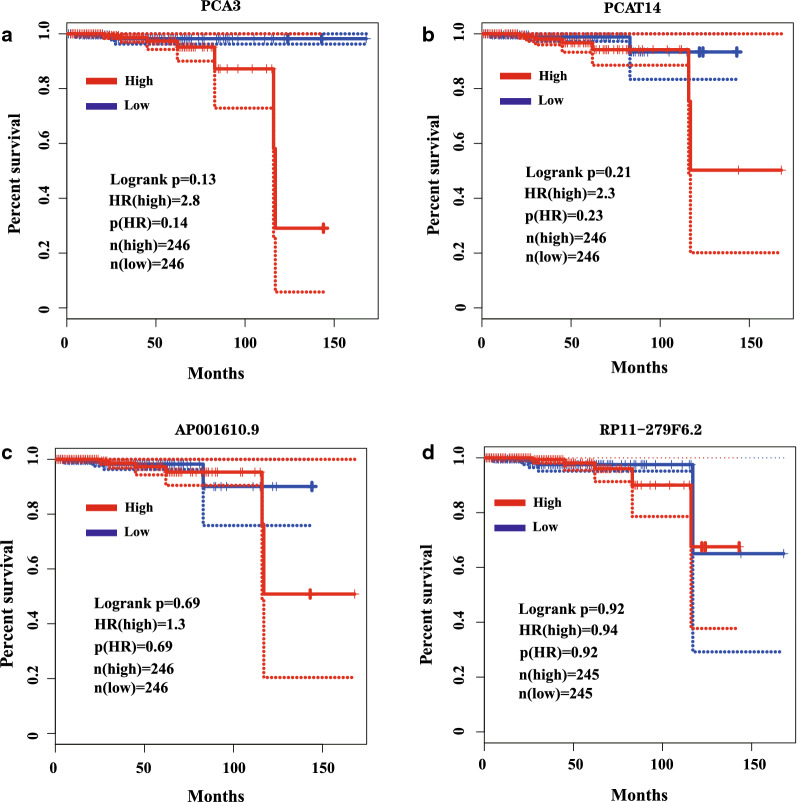
The results of survival curve analysis by GEPIA. **a** The survival curve analysis of PCA3. **b** The survival curve analysis of PCAT4. **c** The survival curve analysis of AP001610.9. (**d**) The survival curve analysis of RP11-279F6.2^*^. ^*^RP11-279F6.2 is the corresponding gene symbol of ENST00000558010 (Table 4)

As the top seven upregulated lncRNAs in our study revealed the coherence of bioinformatics analysis and qPCR analysis, we further analyzed their survival curves of original gene in PCa by the tool of GEPIA (https://gepia.cancer-pku.cn/) (Fig. 6). Higher expression of prostate-specific DD3(PCa3) in patients of PCa showed lower survival rate after about 80 months while the higher expression of PCa associated transcript-14 (PCAT14) demonstrated higher survival rate since approximately 60 months. The high expression of AP001610.9 led to a dramatic decline of survival rate after 110 months despite the phenomenon that it revealed moderately higher survival rate from the 80th to 110th month. Moreover, differentiated expression of RP11-279F6.2 showed a subtle difference that the high expression would result in lower survival rate in the duration of 80th and 105th month. Nevertheless, there was no recorded data of NR_125857, which was the most upregulated lncRNA in our study.

### The co-expression network of lncRNA-miRNA-mRNA

**Fig. 7:**
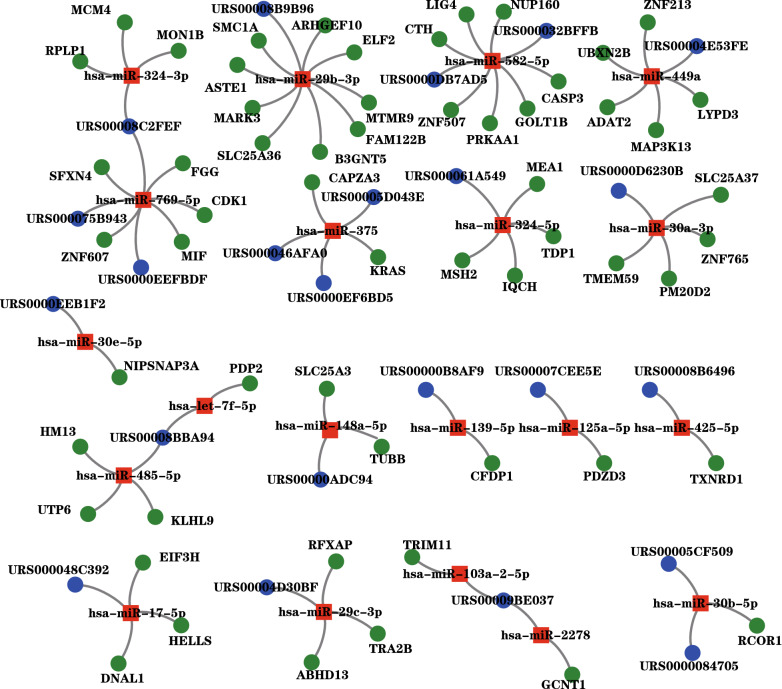
The co-expression network of lncRNA-miRNA-mRNA. In this figure, the red squares represented the miRNAs, the blue circle represented the lncRNAs and the green circles represented the mRNAs

To supplementary achieve perceptions of the lncRNAs’ biological functions in the complex biological processes and cellular regulation, the lncRNA-miRNA-mRNA co-expression network was constructed to investigate the potential interaction between miRNAs, mRNAs and lncRNAs. As shown in the Fig. 7, the co-expression network of lncRNA-miRNA-mRNA included 20 nodes of miRNAs and 84 connections consisting of various lncRNAs and mRNAs. Among the 17 networks, one of the most known co-expression networks was miR-17-5p because it had been proven that miR-17-5p repressed metallopeptidase inhibitor 3 expression in PCa while in this study we found the network of miR-17-5p also got involved in the gene EIF3H, HELLS and DNAL1, which was regulated by the same lncRNA URS000048C392 (also named ENST00000555037.1) [41]. With one edge networks like URS00008B6496(ENST00000547292.1), URS00000B8AF9(ENST00000482003.1), URS0000EEB1F2(ENST00000436764) and URS00007CEE5E(lnc-DHX38-3:6), it should be simple to confirm their roles in PCa by further experiment. Some complicated networks like URS00008C2FEF(ENST00000591956), URS00008BBA94(ENST00000452731) and URS00009BE037(ENST00000492250) were associated with two diverse miRNA signal pathways, which indicated their might have different influence on PCa. URS00005D043E(ENST00000464382), URS000046AFA0(ENST00000534169) and URS0000EF6BD5(ENST00000435802) were connected to the same miR-375, and URS0000DB7AD5(ENST00000580175) and URS000032BFFB(ENST00000558749) were affected by miR-582-5p in the meanwhile. Although with several edges in mRNAs, the rest of lncRNA and miRNAs had the relationship of one–one correspondence. As demonstrated, those lncRNAs, miRNAs and mRNAs were vastly linked as the key hub of the co-expression network, which implied their vitally potential impact on lncRNAs in the progress of regulating particularly target genes in PCa.

## Discussion

We noticed that NR_125857, related to the gene EVADR, ranked the first line of upregulation in our database. EVADR is the written abbreviation of Endogenous retroViral-associated ADenocarcinoma RNA (EVADR), by analyzing RNA-seq data derived from colorectal tumors and matched normal control tissues [42]. This lncRNA demonstrated nominal to low expression in normal tissue, but is significantly upregulated in cancer, particularly in colon, rectal, lung, stomach and pancreas adenocarcinomas. It was reported the EVADR lncRNA determined the promoter activity of the MER48 long terminal repeat (LTR) in vitro, mapped the genome-wide MER48 LTR expression [42]. Regardless of a biological function, the specificity of EVADR activation in adenocarcinomas coupled with the poorer survival probability that tracks with elevated EVADR expression suggested that further characterization of EVADR as a candidate adenocarcinoma biomarker is warranted [42]. Nevertheless, the original article did not mention any details about the EVADR in PCa. In our study, it was totally clear that the expression of NR_125857 is up-regulated in PCa by RNA-seq and qPCR (Fig. 5). Since it was described as the highest upregulated lncRNA in our research, it seemed to be a promising candidate in the further PCa research, for, without any doubt, PCa is also a kind of adenocarcinoma. Because the few investigations in this lncRNA, the co-expression network in this study had not been involved in. The mechanism mediated by the high expression of NR_125857 in PCa requires further cavernous research and clinical following-up.

In the top five of the upregulation in lncRNA, NR_015342 and ENST00000412654 are associated with the PCA3, accounting for a large proportion. PCA3 was located on chromosome 9q21-22 [43]. PCA3, as one of the earliest identified lncRNAs, is an accepted diagnostic urinary biomarker for PCa [44]. Highly overexpression of PCA3 in PCa tissue was found to be a potential non-invasively prediction of prostate biopsy which might be a promising biomarker in clinical diagnosis [45, 46]. In our verification test, we found the consistence of both NR_015342 and ENST00000412654 (Fig. 5). The survival curve also revealed the potential capability of prognostic prediction (Fig. 6a).

Ranking at the third up-regulation of genes, NR_109832 suggests the gene PCAT14 also play an important role in PCa tumorigenesis. PCAT14 is an AR-regulated transcript while PCAT14 is highly expressed in low grade disease and loss of PCAT14 predicts for disease aggressiveness and recurrence, and its overexpression suppresses invasion of PCa cells [47, 48]. PCAT14 lower expression is significantly prognostic for multiple clinical endpoints supporting its significance for predicting metastatic disease that could be used to improve patient management [49]. The outcome of confirmation experiment exhibited the unanimous trend (Figs. 5c, 7b).

The sixth up-regulated gene symbol is related with AP001610.9, and ENST00000415820 may links to TMPRSS2. TMPRSS2, also named as PP9284 or PRSS10, is transmembrane serine protease 2, which is a member of the membrane-anchored serine proteases family [50]. It has been figured out that TMPRSS2 mediates a proteolytic cascade regulated by androgen signaling, which promotes the progression, invasion, and metastasis of PCa cells by activating the matriptase and disordering the extracellular matrix [51, 52]. TMPRSS2 mainly affects degradation of extracellular matrix nidogen-1 and laminin β1 [51]. Therefore, it indicates an innovative approach for targeting these two proteases in treatment development, and the intimate connection between tumor cells and extracellular matrix in the PCa. In our survival curve analysis, the high expression of AP001610.9 led to high hazard ratio after approximately 100 months (Fig. 6c). The relationship between ENST00000415820 and TMPRSS2 would be our interests for research.

The lowest down-regulation lncRNA is the anonymous lnc-MYL2-4:1. In our study, it suggests this lncRNA is interrelated to myosins, which are a large and diverse family of molecular motors important for cell migration and motility [53]. In PCa, Myo1b, Myo6, Myo9b, Myo10, and Myo18a were expressed at higher levels in high metastatic potential cells, and especially Myo1b and Myo10 were expressed at higher levels in metastatic tumors [54–56]. Changes in expression of several myosin isoforms may contribute to metastasis in PCa [54]. The GO analysis in our study showed the enrichment in the muscle system process, muscle hypertrophy and muscle development while KEGG pathway research also revealed vascular smooth muscle contraction got involved in PCa specimens (Fig. 4). Though the outcome of qPCR in this study was no significant different in PCa tissues and normal tissues, the exact interaction between our candidate lncRNA and myosin is still needed to research.

The second down-regulation lncRNA lnc-C19orf73-1:1 is related to histidine rich calcium binding protein (HRC). The HRC is a novel regulator of sarcoplasmic reticulum (SR) Ca^2+^-uptake, storage and release, so the HRC plays a pivotal role in Ca^2+^-homeostasis.2 Calcium (Ca^2+^) is an essential intracellular signaling molecule involved in the regulation of cancer progression, including cell proliferation, apoptosis, invasion and migration [57, 58]. Our KEGG research demonstrated the Calcium signal pathway referred to PCa (Fig. 4). It has been proved that HRC promotes growth of hepatocellular carcinoma in vitro and in vivo [59]. Furthermore, HRC also plays a significant role in myocyte differentiation and in anti-apoptotic cardioprotection against ischemia/reperfusion induced cardiac injury [60]. Intriguingly, the cardiovascular system development, cardiac muscle development, and cardiac muscle hypertrophy were displayed in GO analysis(Fig. 4). We speculated the whole field of muscle, as the part of extracellular matrix component, may make a profound effect on the biological property of PCa (Additional file 1: Table S1).

Lnc-MID1-4:1, located on the chromosome X, is associated with Rho GTPase activating protein 6. Rho GTPases have been figured out to be critical signal transducers, which mediate growth factor-induced changes to the actin cytoskeleton and activating the phagocyte NADPH oxidase [61]. The deleted in liver cancer 1 (DLC-1) gene encodes a GTPase activating protein that acts as a negative regulator of the Rho family of small GTPases, and DLC-1 is assumed as a bona fide tumor suppressor gene in different types of human cancer [62, 63]. Combined the results of our GO analysis, we found the abnormality actin binding in PCa, which hinted that the down-regulation of Lnc-MID1-4:1 might influence on the particularly cellular functions in PCa.

In our analysis, there are ten qualified samples, so our study still has boundedness in the number of samples. To highlight the coherence of our outcomes and practical issues and value, we further extended the clinical samples for qPCR and drew the survival curves of meaningful genes of lncRNAs after the confirmation of qPCR. The top seven upregulation lncRNAs, like NR_125857, NR_015342, NR_109832, ENST00000412654, lnc-AC110080.1–5:1, ENST00000415820 and ENST00000558010 are hopeful research candidates for extra investigation. The present study of lncRNAs in PCa tissues is a proof-of-principle that lncRNAs have a possible character in PCa formation and progression. As demonstrated in the tables, there are so many lncRNAs has the relationship with PCa, so lots of verification test are need to be completed. Since both PCA3 and PCAT14 have been thoroughly studied, so they partly played a special role on the ensuring our research credibility and providing us the reliable reference. With the deep research, the potential mechanism of lncRNA will be disclosed stepwise, which provides new breakthroughs in the early diagnosis, prognosis, and therapy targets of PCa.

## Conclusion

Our study mapped a novel landscape of lncRNA differential expression between PCa and benign prostate tissues. Especially, we first found NR_125857 expression in human PCa tissues was the most up-regulated lncRNA. Moreover, we constructed a co-expression network of lncRNA-miRNA-mRNA for further study of mechanism in PCa. As a promising candidate, further studies are needed to investigate to figure out the mechanisms in PCa.

## Supplementary information

**Additional file 1: Table S1.** Top each 20 up- and down-regulated lncRNAs and corresponding gene information of lncRNAs.

## Data Availability

The datasets used and analyzed in the current study are available from the corresponding author on reasonable request.

## Abbreviations

PCa: Prostate cancer
AR: Androgen receptor
PSA: Prostate specific antigen
CRPC: Castration-resistant prostate cancer
ncRNAs: Non-coding RNAs
tRNAs: Transfer RNAs
rRNAs: Ribosomal RNAs
miRNAs: MicroRNAs
snoRNAs: Small nucleolar RNAs
sRNAs: Small nuclear RNAs
lncRNAs: Long non-coding RNAs
GO: Gene ontology
KEGG: Kyoto Encyclopedia of Genes and Genomes
PCA3: Prostate-specific Dd3
PCAT14: Prostate Cancer Associated Transcript-14
USPSTF: Us preventive services task force
EVADR: Endogenous retroViral-Associated ADenocarcinoma RNA
LTR: Long terminal repeat
GTPases: Guanosine triphosphatases
TMPRSS2: Transmembrane Serine Protease 2
SNORA62: Small Nucleolar RNA, H/Aca Box 62
HRC: Histidine rich calcium binding protein
DLC-1: Deleted in liver cancer 1
