# Supplementary material for: Characterization and validation of long noncoding RNAs as new candidates in prostate cancer
Source: Cancer Cell Int. 2020 Nov 1;20:531. doi: 10.1186/s12935-020-01615-y (PMC7603695; doi:10.1186/s12935-020-01615-y)
Supplement: Supplementary file 1 — Additional file 1: Table S1. Top each 20 up- and down-regulated lncRNAs and corresponding gene information of lncRNAs. [file 12935_2020_1615_MOESM1_ESM.docx]

**Additional Table S1.** Top each twenty up- and down-regulated lncRNAs and corresponding gene information of lncRNAs

| Accession | *P*-values | Fold change | Regulation | Chromosome | | Strand | Gene Symbol | Associated gene description |
| --- | --- | --- | --- | --- | --- | --- | --- | --- |
| NR_125857 | 0.0012796 | 59.276583 | up | | chr6 | + | EVADR | - |
| NR_015342 | 0.003856 | 28.092424 | up | | chr9 | + | PCA3 | prune homolog 2 (Drosophila) |
| NR_109832 | 0.0087905 | 21.726513 | up | | chr22 | + | PCAT14 | - |
| ENST00000412654 | 0.0026089 | 19.731117 | up | | chr9 | + | PCA3 | prune homolog 2 (Drosophila) |
| lnc-AC110080.1-5:1 | 0.0214716 | 17.480829 | up | | chr2 | - | --- | - |
| ENST00000415820 | 0.0175281 | 16.656541 | up | | chr21 | - | AP001610.9 | - |
| ENST00000558010 | 0.0150573 | 15.600412 | up | | chr15 | - | RP11-279F6.2 | - |
| ENST00000365110 | 0.0289324 | 13.988218 | up | | chr11 | + | SNORA62 | - |
| NONHSAT072254 | 0.0075766 | 12.035524 | up | | chr2 | - | --- | - |
| NONHSAT072236 | 0.0072588 | 11.671845 | up | | chr2 | - | --- | - |
| ENST00000439575 | 0.0439698 | 11.653131 | up | | chr10 | + | RP11-118K6.2 | - |
| lnc-AC110080.1-1:1 | 0.0177589 | 11.267734 | up | | chr2 | - | --- | - |
| lnc-AC233264.5-3:1 | 0.0367401 | 10.224691 | up | | chr2 | + | --- | - |
| ENST00000616913 | 0.0034881 | 9.6291064 | up | | chr9 | + | PCA3_1 | prune homolog 2 (Drosophila) |
| NONHSAT072252 | 0.013674 | 9.0948737 | up | | chr2 | - | --- | - |
| lnc-MX1-1:1 | 0.0217773 | 8.6735934 | up | | chr21 | + | --- | - |
| lnc-LRCH4-3:1 | 0.0161447 | 8.2237523 | up | | chr7 | - | --- | ArfGAP with FG repeats 2 |
| lnc-AC110080.1-15:1 | 0.0140691 | 8.1295177 | up | | chr2 | - | --- | - |
| ENST00000621752 | 0.0168726 | 7.9573305 | up | | chr10 | - | RP11-122K13.15 | - |
| lnc-TOMM70A-1:1 | 0.0397496 | 0.2638873 | down | | chr3 | - | --- | filamin A interacting protein 1-like |
| ENST00000623595 | 0.0256375 | 0.2592142 | down | | chr9 | + | RP11-392A14.8 | - |
| lnc-ABHD10-1:1 | 0.0328931 | 0.2574471 | down | | chr3 | + | --- | pleckstrin homology-like domain, family B, member 2 |
| lnc-GADD45B-1:2 | 0.0217582 | 0.2560995 | down | | chr19 | + | --- | guanine nucleotide binding protein (G protein), gamma 7 |
| lnc-C9orf43-5:1 | 0.0179094 | 0.2529903 | down | | chr9 | + | --- | regulator of G-protein signaling 3 |
| lnc-C2CD4A-8:3 | 0.0070292 | 0.2501496 | down | | chr15 | + | --- | RAR-related orphan receptor A |
| lnc-NDRG1-5:1 | 0.0451373 | 0.250131 | down | | chr8 | - | --- | ST3 beta-galactoside alpha-2,3-sialyltransferase 1 |
| lnc-HFM1-3:3 | 0.0435927 | 0.2433965 | down | | chr1 | - | --- | transforming growth factor, beta receptor III |
| ENST00000617916 | 0.0078552 | 0.2319187 | down | | chr19 | - | LLNLR-268E12.1 | - |
| NR_125886 | 0.0124719 | 0.2278954 | down | | chr4 | + | LOC101927636 | - |
| ENST00000623273 | 0.0117887 | 0.2264097 | down | | chr5 | - | CTB-174D11.3 | slit homolog 3 (Drosophila) |
| ENST00000424251 | 0.0143991 | 0.2204195 | down | | chrX | + | RP1-146A15.1 | interleukin 1 receptor accessory protein-like 1 |
| lnc-TACC2-3:1 | 0.0146426 | 0.2146128 | down | | chr10 | + | --- | transforming, acidic coiled-coil containing protein 2 |
| NR_125859 | 0.0322068 | 0.2054874 | down | | chr6 | + | LOC101928540 | filamin A interacting protein 1 |
| NONHSAT136589 | 0.0022274 | 0.1790319 | down | | chrX | - | --- | dystrophin |
| lnc-CHST2-2:3 | 0.023774 | 0.1771025 | down | | chr3 | + | --- | - |
| lnc-PDCD11-5:1 | 0.000183 | 0.1635704 | down | | chr10 | + | --- | neuralized E3 ubiquitin protein ligase 1 |
| lnc-PTEN-11:1 | 0.0032078 | 0.1472261 | down | | chr10 | + | --- | - |
| lnc-MID1-4:1 | 0.00866 | 0.1373993 | down | | chrX | - | --- | Rho GTPase activating protein 6 |
| lnc-C19orf73-1:1 | 0.034911 | 0.030913 | down | | chr19 | - | --- | histidine rich calcium binding protein |
| lnc-MYL2-4:1 | 0.0190929 | 0.0014053 | down | | chr12 | - | --- | myosin, light chain 2, regulatory, cardiac, slow |
